# Need for Cognition Partially Mediates the Relationship Between Cognition and Subjective Well-Being

**DOI:** 10.1093/geroni/igaa057.1985

**Published:** 2020-12-16

**Authors:** Neshat Yazdani, Karen Siedlecki

**Affiliations:** 1 Fordham University, Bronx, New York, United States; 2 Fordham University, New York, New York, United States

## Abstract

Aspects of cognitive functioning have been linked to measures of subjective well-being both cross-sectionally (Jones et al., 2003) and over time (Enkvist et al., 2013) but the mechanisms underlying this relationship remain unclear. One potential mechanism may be individuals’ need for cognition, or the dispositional tendency to enjoy and engage in effortful cognitive activities (Cacioppo & Petty, 1982). Analyses were conducted to examine need for cognition as a mediator of the relationship between five domains of cognition (episodic memory, processing speed, reasoning, spatial visualization, and vocabulary) and four dimensions of subjective well-being (life satisfaction, positive affect, negative affect, and depressive symptomatology) cross-sectionally in a large sample of healthy adults between the ages of 18-99. Results indicate that need for cognition partially mediates the relationship between all five domains of cognition and life satisfaction, negative affect, and depressive symptomatology, but does not mediate the relationship between cognition and positive affect.

